# Genome-Scale Validation of Deep-Sequencing Libraries

**DOI:** 10.1371/journal.pone.0003713

**Published:** 2008-11-12

**Authors:** Dominic Schmidt, Rory Stark, Michael D. Wilson, Gordon D. Brown, Duncan T. Odom

**Affiliations:** 1 Cancer Research UK, Cambridge Research Institute, Li Ka Shing Centre, Cambridge, United Kingdom; 2 Department of Oncology, Hutchison/MRC Research Centre, Cambridge, United Kingdom; Wellcome Trust Sanger Institute, United Kingdom

## Abstract

Chromatin immunoprecipitation followed by high-throughput (HTP) sequencing (ChIP-seq) is a powerful tool to establish protein-DNA interactions genome-wide. The primary limitation of its broad application at present is the often-limited access to sequencers. Here we report a protocol, Mab-seq, that generates genome-scale quality evaluations for nucleic acid libraries intended for deep-sequencing. We show how commercially available genomic microarrays can be used to maximize the efficiency of library creation and quickly generate reliable preliminary data on a chromosomal scale in advance of deep sequencing. We also exploit this technique to compare enriched regions identified using microarrays with those identified by sequencing, demonstrating that they agree on a core set of clearly identified enriched regions, while characterizing the additional enriched regions identifiable using HTP sequencing.

## Introduction

Chromatin immunoprecipitation (ChIP) is widely used to identify interactions between genomic DNA and proteins in eukaryotic cells [1]. Recent approaches have identified transcription factor binding or histone modifications by combining ChIP experiments with genomic-tiling or promoter-based microarrays (i.e. ChIP-chip) [2]–[9]. The subsequent maps of transcription factor binding, chromatin structure, and modifications established by ChIP-chip and similar assays have greatly broadened our understanding of mechanisms regulating transcription [2]–[9]. However, microarrays generally interrogate only genomic regions rich in unique sequences, and their designs typically start by eliminating half of a mammalian genome due to repeat regions.

The application of high-throughput (HTP) DNA sequencing to ChIP experiments (ChIP-seq) has overcome many microarray-related limitations in probe coverage and resolution [10]–[13]. ChIP-seq allows transcription factor and histone patterning of complex mammalian genomes to be identified at high resolution, and the interrogation of virtually an entire mammalian genome is now possible. As HTP sequencing technology can be difficult to access and expensive to use, a method to inspect sequencing library quality and genomic enrichment for ChIP-seq experiments in advance of deep sequencing would be of substantial utility to a wide number of researchers [14].

Here we report such a methodology (Microarrays-before-Sequencing, Mab-seq), which should also be of interest to investigators using chromatin immunoprecipitations who are working primarily with microarrays, but wish to bank material for later genome-wide interrogations. This method also offers advantages to investigators with access to HTP instruments (e.g. Illumina Genome Analyzer) who wish to test their libraries prior to sequencing without compromising either experimental approach by sample loss.

## Results and Discussion

### Adaptation of sequencing libraries for preliminary microarray hybridization

**Figure 1:**
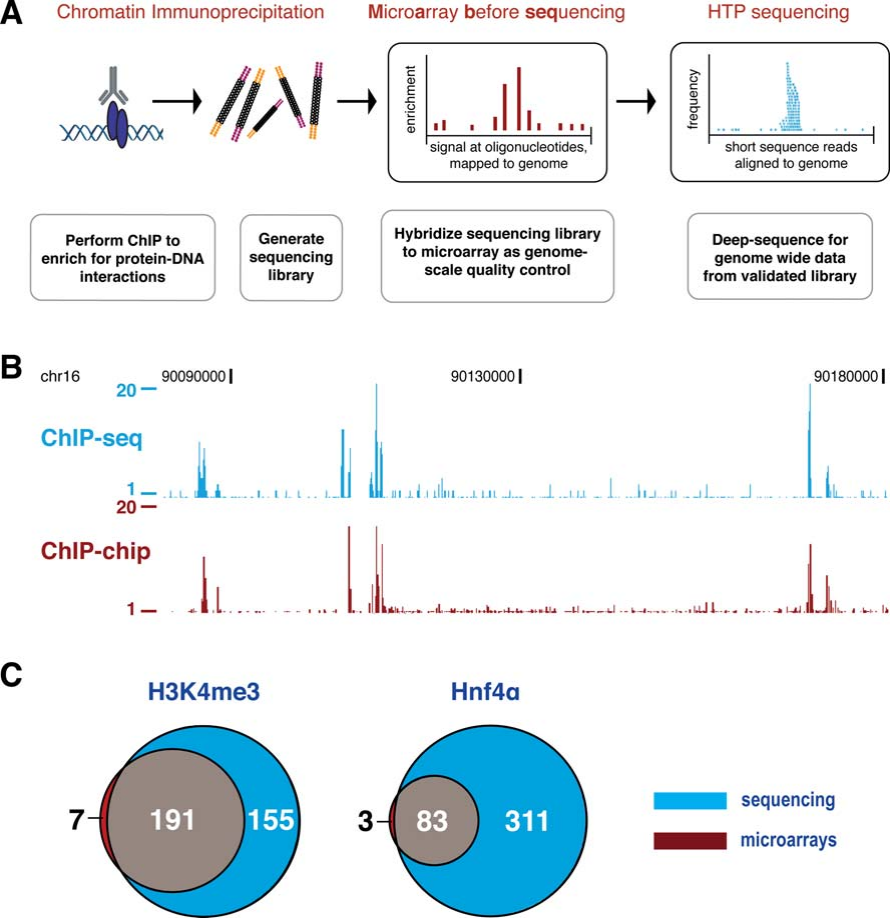
Mab-seq: Microarrays can be used to validate sequencing libraries in advance of deep sequencing. (A) Chromatin immunoprecipitations are performed using standard techniques against either histone marks or site-specific transcription factors, followed by generation of sequencing libraries. A small amount of these libraries are amplified, labeled with fluorophores, and hybridized to commercially available microarrays. After the ChIP signal has been evaluated and passed quality control, the remaining library is deep-sequenced. (B) Direct comparison of Hnf4α ChIP-seq (blue, absolute fragment count) and ChIP-chip (red, ratios for enrichment relative to whole-cell extract) data from the same library across a 100 kb region in mouse hepatocytes. (C) ChIP-chip and ChIP-seq experimental data obtained from the same library show that microarrays accurately predict a subset of sequencing-determined enriched regions, with few enriched regions unique to microarrays, consistent with the greater depth and sensitivity of sequencing technologies.

We developed Microarray-before-sequencing (Mab-seq) for use in ChIP experiments with both transcription factor binding and chromatin modification assays. We modified DNA library preparation for the Illumina Genome Analyzer to allow the small amounts of ChIP-enriched DNA to be simultaneously used on both commercially available microarrays and Illumina sequencers (Figure 1A). This methodology was demonstrated by performing ChIP on hepatocytes isolated directly from mouse liver using well-characterized antisera against the liver-enriched transcription factor Hnf4α [6], [7], [15] and the trimethylated lysine 4 of histone H3 [2], [3], [5], [12]. Because sequence-specific transcription factor ChIPs from primary tissue often show lower enrichment than ChIPs against more general factors such as RNA polymerase II or histone modifications, the use of Hnf4α was an important inclusion in our protocol development.

We remove a small portion of the library preparations and then amplify these aliquots using standard techniques. This material is then labeled with fluorophores, and can be hybridized to any commercially available array. Below, we demonstrate this protocol for ChIP enrichment using mouse chromosome 16 tiling microarrays (Agilent Technologies), followed by subsequent deep sequencing using an Illumina/Solexa 1G Genome Analyzer. This approach can be used with other commercial microarray platforms (Nimblegen, Affymetrix, or self-print microarrays).

### Microarrays accurately predict ChIP enriched regions in HTP sequencing libraries

Comparing microarray probe enrichment to sequencing read depth for the same sample reveals a high degree of correspondence (Figure 1B). Computational identification of enriched regions (enriched regions: ERs, see Methods) confirms this correspondence. Using microarrays, 198 regions were identified as enriched in trimethylation of the lysine 4 position of histone H3 (H3K4me3), of which 191 were also identified by subsequent sequencing (Figure 1C). For the transcription factor Hnf4α, 83 of the 86 ERs present on the microarrays were subsequently identified also by sequencing analysis. Only three Hnf4α microarray bound regions and seven histone-marked regions identified by microarray analysis were not subsequently identified using the corresponding ChIP-seq data.

**Figure 2:**
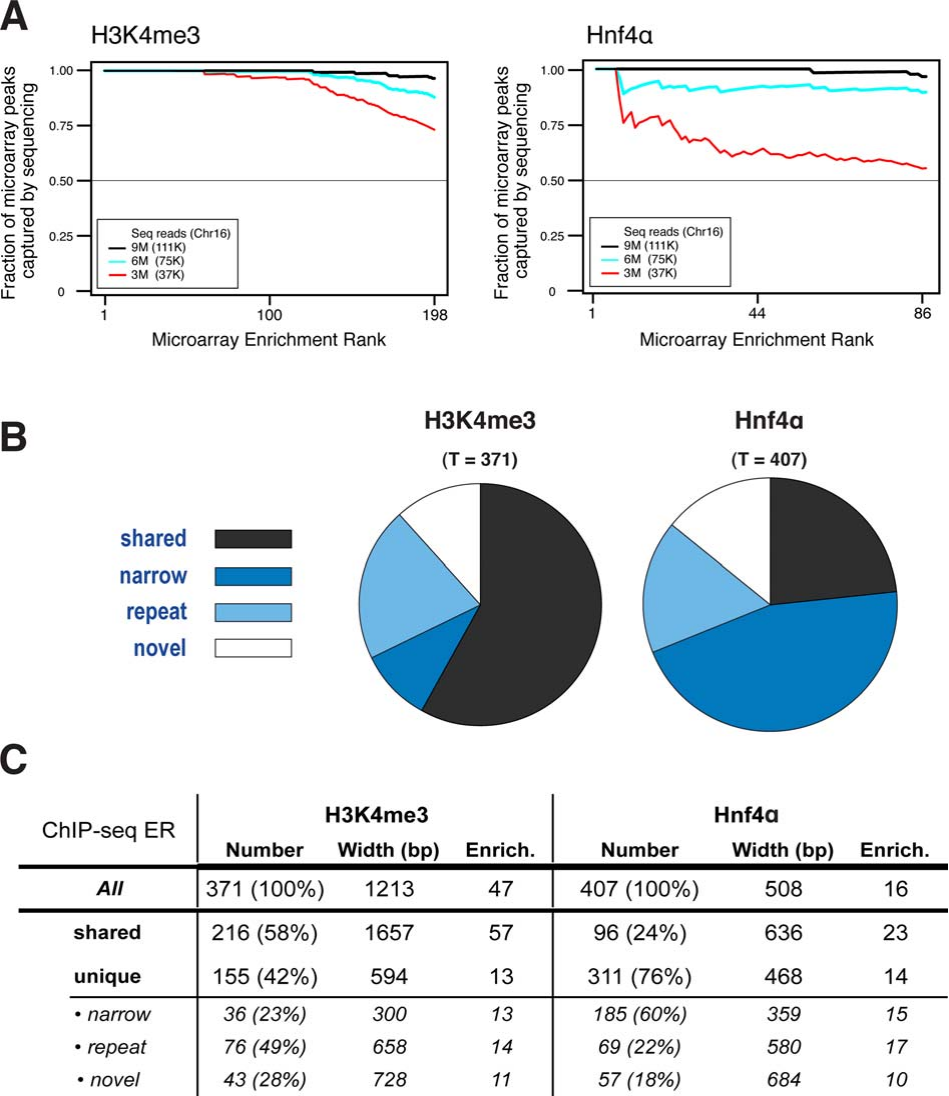
The ability of sequencing to capture the complete set of microarray peaks is critically dependent on the depth of sequencing employed. The cumulative fraction of microarray identified enriched regions captured by a given number of sequence reads is shown as black (9 million), blue (6 million) and red (3 million), with the subset of reads that map to MmChr16, shown in parentheses. (A) Trimethylation of the K4 position of histone H3, mainly found at transcription start sites, has reliably strong enrichment; thus, few reads are needed to identify accurately the microarray-determined H3K4me3 enriched regions. In contrast, Hnf4α, as a site-specific transcription factor, requires substantially greater numbers of reads to capture the microarray determined enriched regions. (B) Proportions of ChIP-seq enrichments that are shared with the microarray (shared) or are ChIP-seq unique: narrow = not enough probes covered; repeat = no probes or too few probes due to repeat masking during microarray probe design; and novel = enough probes covered but microarray signal not above threshold. (C) Table of different ChIP-seq enrichments categories as in B. Number = count of ChIP-seq enrichment regions and the percentage of all (all, shared, unique) or the percentage of unique (narrow, novel, repeat) shown in parentheses; Width = average width in bp; Enrich. = average fold enrichment over input. Note: The numbers of shared ERs differs from the one shown in Figure 1C, because Figure 1C refers to the number of microarray ERs that overlap with sequencing ERs whereas Figure 2C shows the number of sequencing ERs that overlap with microarray ERs. Hence if two sequencing ERs are identified that overlap with a single microarray ER, this will count as one overlapping microarray ER in Figure 1C but as two overlapping sequencing ERs in this Figure (see Methods for an explanation of how overlapping ERs are identified and counted).

Rank ordering of the complete set of bound genomic regions detected by the microarrays versus regions identified as bound by ChIP-seq showed high overlap (Figure 2A, black line). Even modest sequencing of these libraries largely recapitulated microarray results, though Hnf4α shows stronger dependence on depth of sequencing. This dependence appears to be a function of both the narrower region of enrichment found for transcription factors, as well as the lower enrichment for site-specific transcription factors compared to histone mark enrichment. Thus, a sequencing library created from a chromatin immunoprecipitation can be labeled and hybridized to a genomic microarray to accurately and reliably indicate success in ChIP enrichment and library creation, as well as provide a chromosome-scale overview of the eventual deep sequencing information.

Identification of a core set of clearly identified enriched regions serves not only to validate the efficacy of the Mab-seq library preparation protocol, it can also be used as a practical gauge of sequencing progress. Different ChIP libraries will require different quantities of sequencing reads to adequately identify enriched regions (Figure 2A, colored lines). While the total depth of sequencing required may be calculated theoretically based on degree of enrichment [12], determining the degree to which highly enriched regions identified by the microarray have been identified by sequencing reads may provide an empirical check that adequate sequencing depth has been reached.

### Sequencing increases the genomic coverage and sensitivity of chromatin immunoprecipitations

As expected from prior reports, deep sequencing greatly expands the number of identified ERs compared to microarrays by virtue of increased sensitivity and interrogation of repeat-rich regions [16]. Prior studies have compared matched, but not identical, ChIP experiments on microarrays and sequencing [12], [13]. Here, we used the exact same sequencing libraries to identify targets on microarrays and corresponding deep sequencing, providing matched data obtained from these two platforms that are quantitatively comparable.

The enrichment levels of shared ERs (identified by both microarray and sequencing analyses) are much higher than for those identified only by sequencing. Figure 2C shows statistics for different types of ERs, including shared regions identified by both ChIP-chip and ChIP-seq, as well as ERs identified only by ChIP-seq. For the histone modification H3K4me3, shared ERs have a mean enrichment of 57-fold, compared to a mean 13-fold enrichment for sequence-unique ERs (about four-fold increase), while for the transcription factor Hnf4α, the shared ERs have a mean enrichment of 23-fold, compared to 14-fold mean enrichment of sequencing-unique ERs. For H3K4me3, in addition to the 191 ERs shared between microarray and sequencing data, sequencing added an additional 155 ERs that were not detected by microarray analysis (Figure 1C). Similar results are found for the transcription factor Hnf4α, where sequencing uniquely identifies an additional 311 ERs.

The majority of sequencing-unique ERs occur in regions where the microarray does not have sufficient probes to confidently identify enrichment (generally, at least three probes are required). 112 (72%) of the 155 sequencing-unique ERs identified for the histone mark H3K4me3 are found in such regions of the mouse Chr16 (MmChr16) microarray, as are 254 (82%) of the 311 sequencing-unique ERs for the transcription factor Hnf4α. These can be further divided into two subclasses, attributable to two factors: (i) the greater apparent sensitivity of HTP sequencing to ERs spanning smaller numbers of base pairs (‘narrow’ in Figure 2), and (ii) greater genomic coverage, especially in partially-repetitive regions (‘repeat’ in Figure 2).

**Figure 3:**
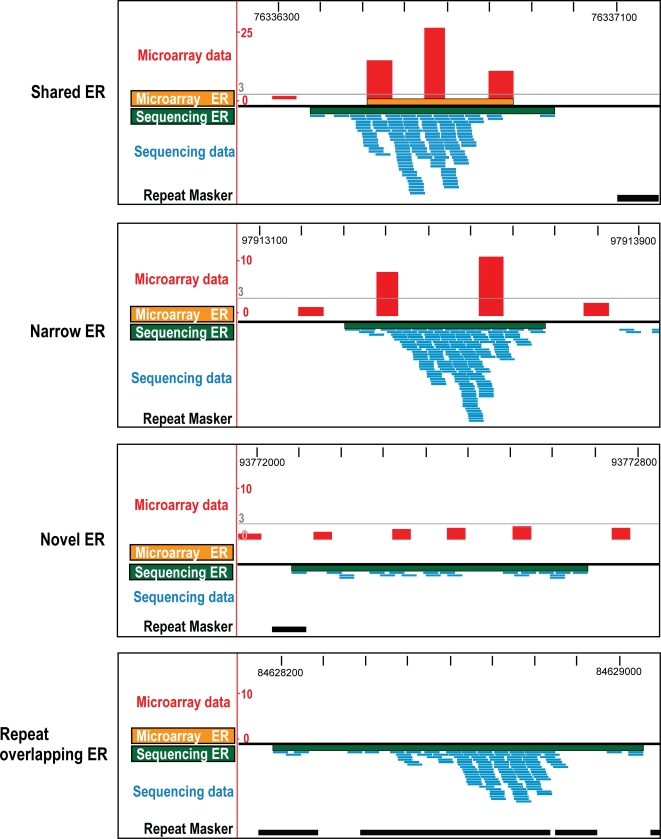
Visualization of matched ChIP-chip and ChIP-seq data for Hnf4α. The four main categories (shared, narrow, novel, and repeat) are described in detail in Figure 2. The x-axis spans 1 kb of mouse chromosome 16 and the y-axis shows the fold enrichment for the microarray data and the depth of sequencing for the sequencing data. The threshold (3-fold) for the microarray-analysis is indicated as a grey line.

The first subclass (‘narrow’) consists of sequencing-unique peaks that are due to the sensitivity limitations inherent in the microarray design. ChIP-chip experiments interrogating ERs of narrow width could be hindered by the combination of microarray design parameters with analytic approaches that require co-voting of spaced probes [2], [6], [7], [9]; for instance, 60 base oligonucleotides with a 200–300 base pair spacing are typical of many chromosomal tiling approaches (e.g. Agilent whole genome microarrays). It is challenging to reliably identify ERs narrower than 400 bases using such a microarray, as such regions will contain two or fewer probes. We found that 36 (23%) of the 155 sequencing-unique ERs identified for H3K4me3 are 400 base pairs or less in width and do not overlap with at least three oligonucleotides present on the Agilent Chr16 microarray, as are 185 (60%) of the 311 sequencing-unique ERs identified for Hnf4α. The greater number of lost peaks for the site-specific transcription factor Hnf4α is likely due to the considerably smaller mean width in bases of enrichment peaks versus H3K4me3 (508 bases versus 1213 bases). Many of these sequencing-unique peaks can be seen as enrichments in the microarray data that span only one or two neighboring probes, too few to confidently identify ERs (see Figure 3). Other commercial microarray platforms (e.g. Nimblegen and Affymetrix) with higher oligonucleotide densities may have less sensitivity to signal fragmentation via probe loss.

For the second subclass (‘repeats’), enrichment in repetitive regions account for 76 (49%) of the 155 sequencing-unique ERs identified for H3K4me3 and 69 (22%) of 311 sequencing-unique ERs for Hnf4α. Many of these previously undetectable enriched regions show high enrichment (Figure 3). Because most commercial microarrays share the early-design requirement of repeat-masking before oligonucleotide selection, the majority of the partially repetitive peaks would have been missed using any oligonucleotide-based microarray.

The few remaining sequence-unique ERs are those for which there is sufficient probe coverage on the microarray, but insufficient sensitivity. The greater sensitivity of deep sequencing enables the identification of ERs with levels of enrichment that, while significant, are too low to be reliably identified via microarray (Figures 2 and 3). These account for 28% of the sequencing-unique ERs for the histone mark H3K4me3 and 18% of the sequencing-unique ERs for the transcription factor Hnf4α.

The Mab-seq protocol allows the reliable detection of transcription factor binding sites and modified histones using a common pool of ChIP-enriched DNA for microarray hybridization and deep sequencing. Mab-seq provides excellent library validation prior to sequencing, and thus can be used to compare different libraries for experimental prioritization. Because ChIP sequencing library creation is comparable in most aspects, including cost, to standard ChIP-chip libraries, our methodology can be used to generate valuable data and a validated collection of libraries in advance of access to a next-generation sequencer. This method can immediately be applied to similar assays such as mapping of DNase hypersensitivity sites, FAIRE and ChIP-SNP [12], [17]–[19]. We have found that the added cost of using proprietary library-building kits, as opposed to previously described ligation mediated PCR and whole genome amplification techniques [4], [8], [20], is somewhat offset by the robust nature of the Illumina library procedure. An additional and important advantage is the time saved in separate sample preparation for distinctly different techniques.

Finally, we have used this technique to compare enriched regions identified using microarrays with those identified by sequencing, demonstrating that they agree on a core set of clearly identified enriched regions that can be used to help gauge sequencing depth in practice. We empirically demonstrate how, using the same ChIP library, sequencing enables the identification of a larger set of enriched regions, including regions with low density of microarray probe coverage due to the narrowness of the region of enrichment or the presence of repetitive elements that prevent unique oligonucleotides from being placed on the microarray.

In summary, Mab-seq combines the relative ease and low-cost of microarrays with the power and depth of HTP sequencing to create flexible, quality-tested libraries that can be archived and re-used multiple times.

## Methods

Antibodies used: Hnf4α (sc-8987, Santa-Cruz) and H3K4me3 (ab8580, Abcam).

Data accession number at ArrayExpress: E-TABM-485 (ChIP-chip). Data accession number at NCBI Short Read Archive: SRA001097 (ChIP-seq). The detailed Mab-seq experimental protocol is included as a supplemental file (Methods S1). This includes the detailed procedures for ChIP and library generation for microarray hybridization and deep sequencing.

### ChIP-chip microarray hybridization

Briefly, 1 µg of a reamplified ChIP-seq library was labeled with Cyanine 5-dUTP and the input control was labeled with Cyanine 3-dUTP (Enzo life sciences) using BioPrime Array CGH Genomic Labeling System kit following the manufacturer's protocol. Unincorporated dyes were removed using QIAquick PCR clean-up kit. Equal amounts of Cy5 and Cy3 labeled DNA was combined and hybridized at 65°C to microarrays using 2× Hi-RPM Hybridization Buffer Gene expression and manufacturer's protocols. After 40 hours hybridization, arrays were washed with Agilent Array CGH wash buffers 1 and 2 following the manufacturer's protocol and scanned using the Agilent scanner. Raw data was extracted using the Agilent Feature Extraction Software, and processed as mentioned below.

### ChIP-chip microarray analysis

The statistical analysis was performed using the *limma* package. Quality of the arrays was assessed using array images of the background and foreground intensities and also of the red/green (Cy5/Cy3) ratios to check for spatial artifacts. A region was considered enriched if it consisted of at least four probes, none of which were more than 600 bases apart, with at least 66% of the probes exhibiting enrichment over threshold. The threshold was set at 5 for the H3K4me3 sample, and 3 for the Hnf4α sample. These thresholds were determined empirically to enable the closest correspondence to the ChIP-seq results. Additionally, regions were enriched if they were not part of another enriched region and consisted of either three probes (none more than 600 bases apart), all of which were over the enrichment threshold, or of two probes less than 600 bases apart, both over the enrichment threshold but with no other probes within 600 bases. Base addresses for the microarrays are based on the NCBI Build 36/mm8 (February 2006) assembly of the *Mus musculus* genome.

### Identification of enriched regions using sequence data

Sequences were aligned to the NCBI Build 37/mm9 (July 2007) assembly of the *Mus musculus* genome using Illumina Genome Analyzer Pipeline Version 0.3.0 (Eland). Approximately 98% of the sequences used in the analysis aligned uniquely with zero, one, or two mismatches in the first 32 bases (see Table S1). The number of aligned sequences for the input sample was 6,660,659 of which 89,064 aligned to the portion of mouse chromosome 16 region covered by the microarray. For each treatment sample, 8,904,321 aligned reads were used, corresponding to 111,224 sequence reads in the region covered by the microarray. The aligned sequences were converted to coordinates on the NCBI Build 36/mm8 (February 2006) genome assembly using the DBAdaptor interface to Ensembl. Enriched regions were identified according to a procedure similar to that used by [11]. A region was considered enriched if it contained at least 10 sequences, none separated by more than 100 bases, and the total number of sequences in the region exhibited at least a 5-fold enrichment over the number of sequences seen in the same region from an input run. The set of enriched regions identified did not vary considerably over a range of parameter settings.

### Comparison and ranking of enriched regions

ERs were ranked by their mean enrichment values (mean fold enrichment of probes for microarray-identified ERs, and fold enrichment over input for sequencing-identified ERs). Using these enrichment scores, we observed a greater dynamic range for common enriched regions measured by sequencing over those measured using microarrays (Figure S1).

Enriched regions identified using different techniques (microarray and sequencing) were considered to be shared if they overlapped at least one base. For comparison purposes, overlapping ERs were merged into single extended regions in a manner similar to that discussed in [21]. In some cases, these merged regions encompass differing numbers of sequencing-identified and microarray-identified ERs, leading to the different numbers reported for microarray-identified ERs also identified by sequencing versus sequencing-identified ERs also identified by microarray.

## Supporting Information

Methods S1 Detailed Mab-seq Protocol for ChIP-seq and ChIP-chip of the same library
(0.20 MB DOC)

Table S1 Mapping statistics of the sequencing reads used in this study
(1.33 MB TIF)

Figure S1 ChIP-seq exhibits greater effective dynamic range than ChIP-chip. Enrichment scores for matched ERs are plotted using the mean enrichment on the microarray and the fold enrichment over Input for UHTP sequencing. The red lines indicate a linear regression, with the Pearson correlation coefficient also reported. (A) Enrichment values of ERs for trimethylation of the K4 position of histone H3 have a more linear relationship (R = 0.68), with the effective fold range for microarrays ranging from 0 to 50, while enrichment found by sequencing extend to 300-fold. (B) Range of enrichment values for Hnf4a is more restricted, with a maximum of approximately 18-fold on the microarray but still extended above 100-fold using UHTP sequencing. Correlation of enrichment values for Hnf4a using the two methods is relatively low (R = 0.51) compared to H3K4me3.
(2.07 MB TIF)
