# Supplementary material for: Genome-Scale Validation of Deep-Sequencing Libraries
Source: PLoS One. 2008 Nov 12;3(11):e3713. doi: 10.1371/journal.pone.0003713 (PMC2577887; doi:10.1371/journal.pone.0003713)
Supplement: Methods S1 — Detailed Mab-seq Protocol for ChIP-seq and ChIP-chip of the same library (0.20 MB DOC) [file pone.0003713.s001.doc]

**Genome-Scale Validation of Deep-Sequencing Libraries**

Dominic Schmidt^†^, Rory Stark^†^, Michael D. Wilson, Gordon D. Brown, Duncan T. Odom
**_____________**

**SUPPLEMENTAL METHODS**

# Mab-seq Protocol

# I. Formaldehyde cross-linking of solid liver tissue

Mice primarily of the 129S8 x C57BL6/J strain [1] were sacrificed at an age of 2 months and the livers were harvested:

1. Isolate livers by terminal anaestesia and rapid 1x PBS perfusion.

Mince livers. Add a 1/10 volume of fresh 11% formaldehyde solution (recipe below) to flasks containing minced tissues.

1. Swirl flasks briefly and let them sit at RT for 20 min. Tumbling is acceptable, though once equilibrated not necessary.
2. Add 1/20 volume of 2.5 M glycine to quench formaldehyde, and allow to sit 10 minutes.
3. Rinse minced tissues twice with 5 ml 1X PBS or 1x Hank’s Balanced Saline.
4. Transfer tissue to 15ml conical tubes using 1x PBS or 1x HBSS and pellet tissue at 4°C.
5. Flash-freeze pelleted tissue in liquid nitrogen or dry ice, and store pellets at –80°C until convenient shipping.

***Formaldehyde Solution***

Final Conc. Stock **For 50 ml**

50 mM 1M Hepes-KOH, pH 7.5 2.5 ml

100 mM 5M NaCl 1.0 ml

1 mM 0.5M EDTA 100.0 µl

0.5 mM 0.5M EGTA 50.0 µl

11% 37% Formaldehyde 14.9 ml

ddH_2_O 31.5 ml

# *Glycine Solution (MW= 75.07 Da)*

Final Conc. Stock For 100 ml

# 2.5 M Glycine (solid) 18.77 g

# ddH_2_O ad 100 ml

# II. Preparing hepatocytes for nuclear lysis

1. Frozen crosslinked material from -80 freezer is allowed to thaw on ice.
2. Tissue is dounced in ice-cold PBS with the loose and the tight plungers (we don’t use protease inhibitors at this step but you could).
3. Dounced tissue is filtered into a 50 ml tube through a 100 um cell strainer. If necessary, the top of a plunger from a 10ml disposable syringe (i.e. where your thumb goes) is used to break up clumps and helps increase your yield. The douncer and filter are rinsed with PBS making up to 40 ml volume. Centrifuge at 4 ºC at 2500 x g for 3 min.
4. Pour off supernatant and repeat wash.
5. Resuspend pellet in 10 ml ice cold PBS and transfer to 15 ml tube, centrifuge as above.
6. Cells now ready for lysis or can be frozen at -80 ºC.

# III. Preblock and binding of antibody to magnetic beads

NOTE: Exact type of Dynal bead (Protein A, Protein G, sheep anti-mouse IgG, sheep anti-rabbit IgG, etc.) depends on antibody being used.

1. Add 100 µl Dynal magnetic beads to microfuge tube. Add 1 ml block solution. Set up 1 tube per IP.
2. Collect the beads using magnetic stand. Remove supernatant.
3. Wash beads in 1.5 ml block solution two more times.
4. Resuspend beads in 250 µl block solution and add 10 µg of antibody.
5. Incubate overnight or at least 4h on a rotating platform at 4°C.
6. Next day, wash beads as described above (3 times in 1 ml block solution).
7. Resuspend in 100 µl block solution.

***Block Solution***

Final Conc. Stock For 50 ml

0.5% BSA (w/v) BSA 250.0 mg

# 1x PBS up to 50.0 ml

# 50.0 ml

**VI. Cell Sonication**

1. Resuspend each pellet from step II (one mouse liver) in 10 ml of LB1. Rock at 4°C for 10 min. Spin at 1,350 x g for 5 minutes at 4°C in a tabletop centrifuge.
2. Resuspend each pellet in 10 ml of LB2. Rock gently at 4ºC for 5 min. Pellet nuclei in tabletop centrifuge by spinning at 1,350 x g for 5 minutes at 4°C.
3. Resuspend each pellet in each tube in 3 ml LB3.
4. Transfer cells to a homemade “sonication tube” (cut a polypropylene, 15ml conical tube into two pieces at the 7 ml mark).
5. Sonicate suspension with a microtip attached to Misonix 3000 sonicator. Samples should be kept in an ice water bath during sonication. Sonicate 8 - 12 cycles of 30 sec ON and 60 sec OFF. Output should be at least 27 Watt. [NOTE: The optimal fragment size for sequencing is about 200 – 300 bp. However it is sufficient if the majority of the fragments have this size.]
6. Add 300 µl of 10% Triton X-100 to sonicated lysate. Split into 2 ml centrifuge tubes. Spin at 20,000 x g for 10 minutes at 4°C to pellet debris.
7. Combine supernatants from the 2 ml centrifuge tubes in a new 15 ml conical tube fill up to 9 ml with LB3 and 600 µl of 10% Triton X-100 for immuoprecipitation. Mix and split into three 15 ml conical tubes, so that each contains 3 ml of cell lysate with 300 µl Triton per ChIP.

8. Save 50 µl of cell lysate from each sample as WCE DNA. Store at -20°C.

NOTE: Add protease inhibitors (final concentration 1x) to all lysis buffers before use.

(Dissolve one Complete Tablet (Roche) in 1 ml H_2_O to make a 50x solution.)

***Lysis Buffer 1 (LB1****)*

Final Conc. Stock For 100 ml

50 mM 1M Hepes-KOH, pH 7.5 5.0 ml

140 mM 5M NaCl 2.8 ml

1 mM 0.5M EDTA 0.2 ml

10% 50% glycerol 20.0 ml

0.5% 10% NP-40 5.0 ml

0.25% 10% Triton X-100 2.5 ml

ddH_2_O 64.5 ml

***Lysis Buffer 2 (LB2****)*

Final Conc. Stock For 100 ml

10 mM Tris-HCl, pH 8.0 1.0 ml

200 mM 5M NaCl 4.0 ml

1 mM 0.5M EDTA 0.2 ml

0.5 mM 0.5M EGTA 0.1 ml

ddH_2_O 94.7 ml

***Lysis Buffer 3 (LB3****)*

Final Conc. Stock For 100 ml

10 mM Tris-HCl, pH 8.0 1.0 ml

100 mM 5M NaCl 2.0 ml

1 mM 0.5M EDTA 0.2 ml

0.5 mM 0.5M EGTA 0.1 ml

0.1% 10% Na-Deoxycholate 1.0 ml

0.5% 20% N-lauroylsarcosine 2.5 ml

ddH_2_O 93.2 ml

**V. Chromatin immunoprecipitation**

1. Add 100 µl antibody/magnetic bead mix to cell lysates.

2. Gently mix overnight on rotator or rocker at 4°C

# VI. Wash, elution, and cross-link reversal

# Steps 1 through 6 should be done in a 4°C cold room.

1. Pre-chill one 1.5 ml microfuge tube for each IP.
2. Transfer half the volume of an IP to a pre-chilled tube.
3. Let tubes sit in magnetic stand to collect the beads. Remove supernatant and add remaining IP. Let tubes sit again in magnetic stand to collect the beads
4. Add 1 ml Wash Buffer (RIPA) to each tube. Remove tubes from magnetic stand and shake or agitate tube gently to resuspend beads. Replace tubes in magnetic stand to collect beads. Remove supernatant. Repeat this wash 4-6 more times.
5. Wash once with 1 ml TE that contains 50 mM NaCl or TBS.
6. Spin at 960 x g for 3 minutes at 4°C and remove any residual TE buffer.
7. Add 200 µl of elution buffer.
8. Elute and reverse crosslink at 65°C for 6-18 h. Resuspend beads in the first 15 min with brief vortexing every five minutes.
9. Spin down beads at 16,000 x g for 1 minute at room temperature.

# Thaw 50 µl of WCE reserved after sonication, add 150 µl (3 volumes) of elution buffer and mix. Reverse crosslink of this WCE DNA by incubating at 65°C overnight.

***Wash Buffer (RIPA)***

Final Conc. Stock For 250 ml

50 mM 1M Hepes-KOH, pH 7.6 12.5 ml

500 mM 5M LiCl 25.0 ml

1 mM 0.5M EDTA 0.5 ml

1% 10% NP-40 25.0 ml

0.7% 10% Na-Deoxycholate 17.5 ml

# ddH_2_O 169.5 ml

***Elution Buffer***

Final Conc. Stock For 100 ml

50 mM 1M Tris-HCl, pH 8.0 5.0 ml

10 mM 0.5M EDTA 2.0 ml

1% 10% SDS 10.0 ml

# ddH_2_O 83.0 ml

**VII. Digestion of cellular protein and RNA**

1. Remove 200 µl of supernatant and transfer to new tube.

2. Add 200 µl of TE to each tube of IP and WCE DNA to dilute SDS in elution buffer.

3. Add 8 µl of 1 mg/ml RNaseA (Ambion Cat # 2271).

4. Mix and incubate at 37°C for 30 min.

5. Add 4 µl of 20 mg/ml proteinaseK (Invitrogen).

6. Mix and incubate at 55°C for 1-2 hours.

7. Add 400ul phenol:chloroform:isoamyl alcohol (P:C:IA) and separate phases with 2 ml Heavy Phaselock tube (follow instructions provided by Eppendorf).

8. Transfer aqueous layer to new centrifuge tube containing 16 µl of 5M NaCl (200 mM final concentration) and 1 µl of 20 µg/µl glycogen.

1. Add 800 µl EtOH. Incubate for 30 min at -80°C.
2. Spin at 20,000 x g for 10 minutes at 4°C to pellet DNA. Wash pellets with 500 µl of 80% EtOH.
3. Dry pellets and resuspend each in 30 µl of 10mM Tris-HCl, pH 8.0.
4. Measure DNA concentration of WCE with NanoDrop (NanoDrop Technologies). Do not measure the ChIP sample.

**VIII. Perform End Repair, add “A” Bases to 3’ Ends of the DNA**

**Important Notes:**

**Steps A-D require reagents from the Genomic DNA sequencing kit by Illumina. If you want to validate your ChIPseq library by means of microarray or qPCR, you must also process an Input sample! In general this is also recommended when you perform ChIPseq only.**

# A. End Repair

# Pipett the following mix direct into PCR tubes and keep on ice.

ChIP sample or 50 ng of Input 30.0 µl

Water 45.0 ul

T4 DNA Ligase buffer with 10 mM ATP 10.0 µl

dNTP mix (each 10 mM) 4.0 µl

T4 DNA polymerase 5.0 µl

Klenow DNA polymerase 1.0 µl

T4 PNK 5.0 µl

Total 100.0 µl

1. Incubate 30 min at 20 ºC in a thermal cycler.
2. Cleanup samples using the DNA Clean&Concentrator-5 kit, (Zymo research,USA), following the manufacturer’s protocol.
3. Elute with 32 µl EB preheated to 50 ºC. Chill on ice.

**B.** **Add “A” Bases**

# Pipett the following mix direct into PCR tubes and keep on ice.

DNA sample 32.0 µl

Klenow buffer 5.0 ul

dATP (1mM) 10.0 µl

Klenow (3’-5’ exo minus; 5 U/µl) 3.0 µl

Total 50.0 µl

1. Incubate 30 min at 37 ºC in a thermal cycler.
2. Cleanup samples using the DNA Clean&Concentrator-5 kit, (Zymo research,USA), following the manufacturer’s protocol.
3. Elute with 8 µl EB preheated to 50 ºC. Chill on ice.

**C.** **Ligate sequencing Adapters to DNA fragments**

# Pipett the following mix in 1.5 ml tubes.

DNA sample 8.0 µl

DNA Ligase buffer 12.5 ul

Adapter Oligo mix (from Illumina for Solexa sequencing) 2.0 µl

DNA ligase 2.5 µl

Total 25.0 µl

1. Incubate 15 min at RT. (Requires Rapid DNA Ligase, else incubate 16 h at 16 ºC.
2. Cleanup samples using the DNA Clean&Concentrator-5 kit, (Zymo research,USA), following the manufacturer’s protocol.
3. Elute with 23 µl EB preheated to 50 ºC. Chill on ice.

**D. Enrich Adapter-Modified DNA by PCR:**

# Pipett the following mix direct into PCR tubes and keep on ice.

DNA sample 23.0 µl

Phusion DNA pol 2x master mix 25.0 ul

PCR primer 1.1 1.0 µl

PCR primer 2.1 1.0 µl

Total 50.0 µl

1. Run program “SOLEXA”:

Step1: 98°C 30 sec

Step2: 98°C 10 sec

Step3: 65°C 30 sec

Step4: 72°C 30 sec

Step5 GOTO Step2 17 times

Step6: 72°C 5 min

Step7: 4°C HOLD

1. Purify with QIAquick and elute with 33,5 µl preheated EB.
2. Take 1 µl of the eluted DNA and mix with 9 µl EB and store as Solexa18. The reminder is stored as SolexaPreGel.

**NOTE:** Solexa18 is the aliquot, which is used for reamplification and microarray or real time PCR analysis. SolexaPreGel is the sample, which is ready for sequencing after Gel purification.

**IXa. Reamplification of Solexa18 for ChIPchip or ChIP-qPCR.**

1. Mix 2 µl of Solexa18 samples in PCR tubes (Do not forget the Input).
2. Make PCR mix:

#### Mix:

Stock 1x Mix

10X Thermopol buffer (NEB) 5.0 µl

dNTP mix (25 mM each) 0.5 µl

Primer 1.2 for reamp[[1]](#footnote-2) (10 µM) 2.5 µl

Primer 2.2 for reamp[[2]](#footnote-3) (10 µM) 2.5 µl

AmpliTaq 1.0 µl

ddH_2_O 36.5 µl

48.00 µl

1. Add 48 ul of Mix A to each sample and run program “REAMP”:

Step1: 95°C 2 minutes

Step2: 95°C 30 sec

Step3: 65°C 30 sec

Step4: 72°C 1 minute

Step5: GOTO Step2 24 times

Step6: 72°C 5 minutes

Step7: 4°C HOLD

1. After PCR is completed, cleanup samples with QIAquick minelute PCR Purification Kit. Elute with 25 µl EB.
2. Measure DNA concentration of all samples with NanoDrop (NanoDrop Technologies).
3. Samples are ready for further processing towards microarray or real-time PCR.

**IXb. Gel purification of SolexaPreGel for ChIPseq**

1. Cast a 50 ml 2 % agarose (BIO-RAD cat#161-3106) TAE gel with 1x SybrSafe.
2. Add 3 µl of 50 % glycerol to 8 µl of DNA ladder (NEB cat# N3233L).
3. Add 10 µl of 50 % glycerol to SolexaPreGel samples from part VIII D.
4. Load all of the ladder into the first well of the gel, leave one lane empty and load the SolexaPreGel sample into the next well. (If you have multiple samples you have to prepare one Gel for each! Do not run more then one sample per gel! Do not cut gels in half!)
5. Run gel at 120 V for 45 min.
6. Excise the 200 – 300 bp fragments on a Dark Reader and purify the DNA with a Qiagen MinElute Gel Extraction Kit. Elute with 15 µl EB preheated to 50 ºC. You can store the larger fragments (300 – 800 bp) as backup.
7. Run the library on a Bioanalyzer to estimate the concentration.
8. Sequence the sample….have fun with the data.


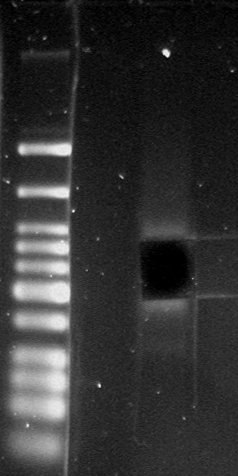


**Supplemental References:**

1. O'Doherty A, Ruf S, Mulligan C, Hildreth V, Errington ML, et al. (2005) An aneuploid mouse strain carrying human chromosome 21 with Down syndrome phenotypes. Science 309: 2033-2037.

1. Order the following primer:

   Primer 1.2 for reamp: 5'AATGATACGGCGACCACCGAGATCTACACTCTTTCCCTACACGACGCTCTTCCGATCT [↑](#footnote-ref-2)
2. Order the following primer:

   Primer 2.2 for reamp: 5' CAAGCAGAAGACGGCATACGAGCTCTTCCGATCT

   Oligonucleotide sequences -- reference http://intron.ccam.uchc.edu/groups/tgcore/wiki/013c0/Solexa_Library_Primer_Sequences.html [↑](#footnote-ref-3)
