# Supplementary figures and images for: Genome-Scale Validation of Deep-Sequencing Libraries
Source: PLoS One. 2008 Nov 12;3(11):e3713. doi: 10.1371/journal.pone.0003713 (PMC2577887; doi:10.1371/journal.pone.0003713)

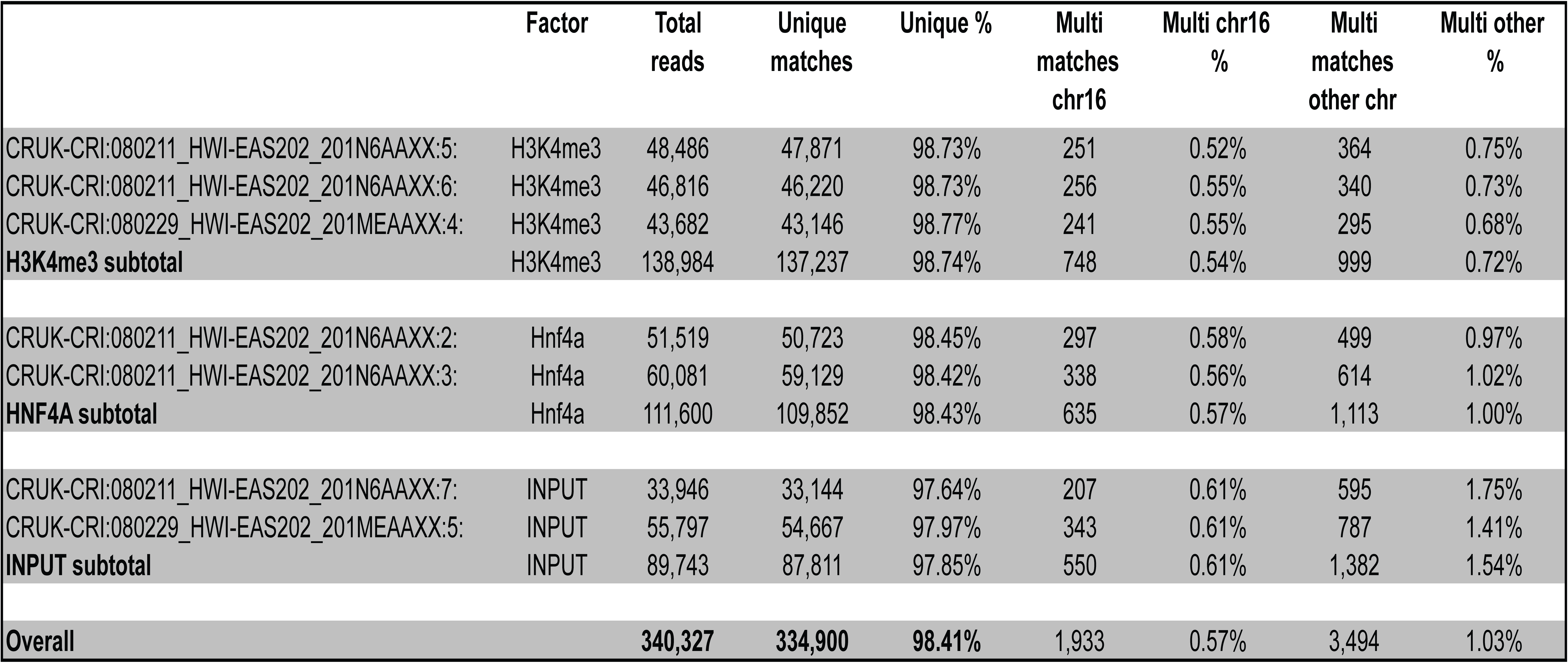

Supplement: Table S1 — Mapping statistics of the sequencing reads used in this study (1.33 MB TIF) [file pone.0003713.s002.tif]

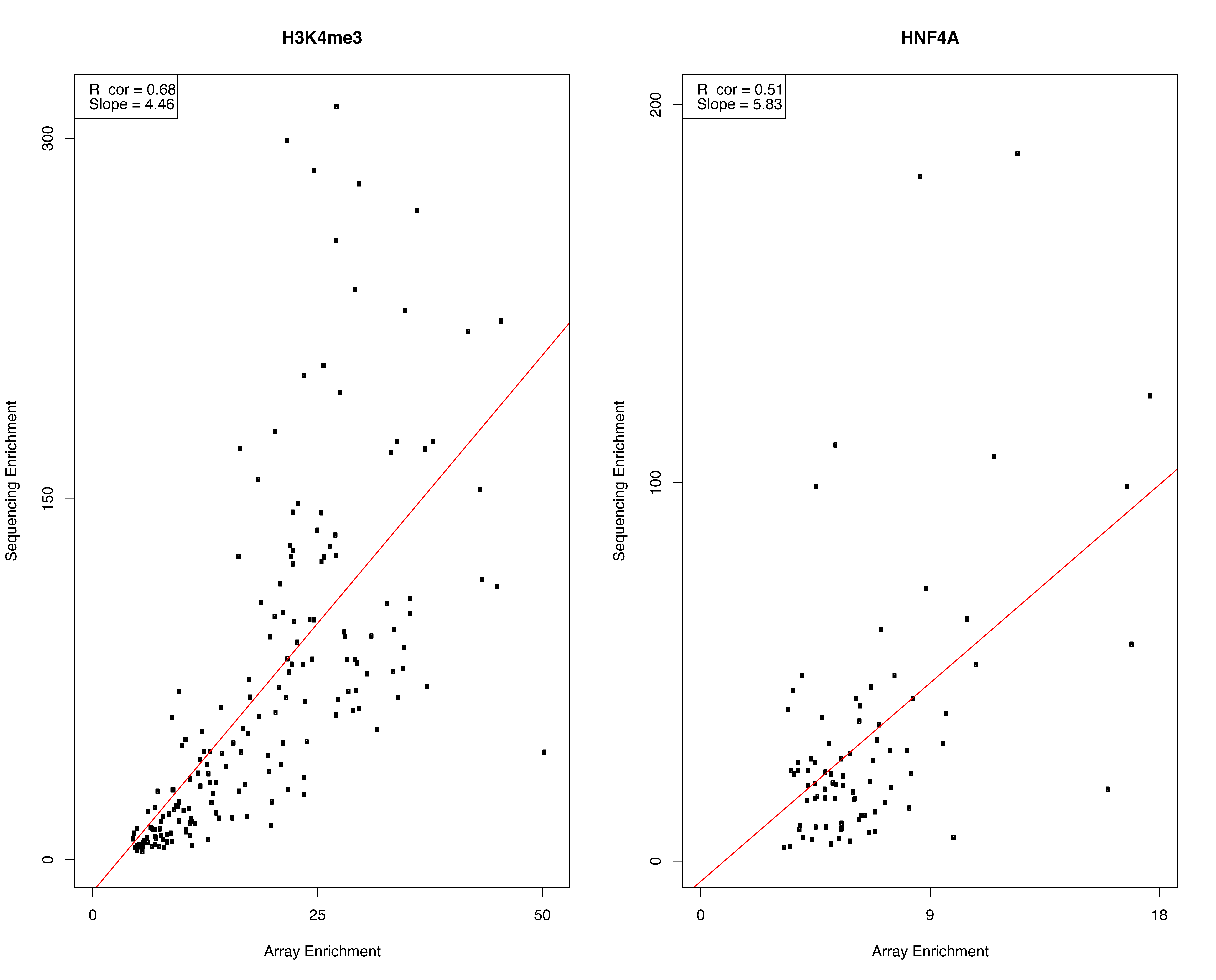

Supplement: Figure S1 — ChIP-seq exhibits greater effective dynamic range than ChIP-chip. Enrichment scores for matched ERs are plotted using the mean enrichment on the microarray and the fold enrichment over Input for UHTP sequencing. The red lines indicate a linear regression, with the Pearson correlation coefficient also reported. (A) Enrichment values of ERs for trimethylation of the K4 position of histone H3 have a more linear relationship (R = 0.68), with the effective fold range for microarrays ranging from 0 to 50, while enrichment found by sequencing extend to 300-fold. (B) Range of enrichment values for Hnf4a is more restricted, with a maximum of approximately 18-fold on the microarray but still extended above 100-fold using UHTP sequencing. Correlation of enrichment values for Hnf4a using the two methods is relatively low (R = 0.51) compared to H3K4me3. (2.07 MB TIF) [file pone.0003713.s003.tif]
